# Acceptability and timing considerations when administering patient-reported outcome measures (PROMs) among people with chronic health conditions who are culturally and linguistically diverse (CALD): a qualitative study protocol

**DOI:** 10.1136/bmjopen-2023-083346

**Published:** 2024-09-12

**Authors:** Jessica Nikolovski, Rachael L. Morton, Rebecca Mercieca-Bebber, Matilda Armstrong, Gill Hartas, Brad Rossiter, Margaret Fagan, Melissa Tinsley, Claire Snyder, Olalekan Lee Aiyegbusi, Rubina Amin-Korim, Kim Sutherland, Claudia Rutherford

**Affiliations:** 1 NHMRC Clinical Trials Centre, Camperdown, New South Wales, Australia; 2 The University of Sydney Susan Wakil School of Nursing and Midwifery, Sydney, New South Wales, Australia; 3 NSW Agency for Clinical Innovation, St Leonards, New South Wales, Australia; 4 Departments of Medicine, Oncology, and Health Policy & Management, Johns Hopkins School of Medicine, Baltimore, Maryland, USA; 5 Centre for Patient-Reported Outcomes Research (CPROR), University of Birmingham Institute of Applied Health Research, Birmingham, UK; 6 Clinical Innovation and Research Division, New South Wales Office for Health and Medical Research, St Leonards, New South Wales, Australia; 7 Sydney Quality of Life Office (SQOLO), The University of Sydney Susan Wakil School of Nursing and Midwifery, Sydney, New South Wales, Australia

**Keywords:** Patient Reported Outcome Measures, Chronic Disease, QUALITATIVE RESEARCH, Health Surveys

## Abstract

**Abstract:**

**Introduction:**

Patient-reported outcome measures (PROMs) are validated and standardised questionnaires that capture patients’ own reports of their symptoms, functioning and well-being. PROMs can facilitate communication between patients and clinicians, reduce symptom burden, enhance quality of life and inform health service re-design. We aim to determine the acceptability of PROMs and the preferred timing of PROM completion in New South Wales (NSW) at the point of care, facilitated by the Health Outcomes and Patient Experiences (HOPE) platform.

**Methods and analysis:**

Semi-structured interviews with patients (~50-75, sampling across seven language groups and seven clinical cohorts), carers (~10–20) and clinicians (~18) enrolled in HOPE will be conducted via videoconference, telephone or in person. Participants will be asked questions about (1) what makes PROMs acceptable for use in chronic disease management (2) when patients would prefer to complete PROMs and when clinicians would like to use PROMs for clinical decision-making and (3) factors that impede the acceptability of PROMs for culturally and linguistically diverse patients. Interviews will be analysed using a reflexive thematic approach, guided by Normalisation Process Theory.

**Ethics and dissemination:**

Ethics approval has been obtained from the Sydney Local Health District Human Research Ethics Committee (SLHD HREC, Study Protocol #X24-0138). Results will be published in appropriate peer-reviewed journals, presented at conferences, disseminated to participants in the form of a plain language summary, and widely disseminated to consumer groups and professional stakeholders.

**STRENGTHS AND LIMITATIONS OF THIS STUDY**

- We will focus on culturally and linguistically diverse communities in New South Wales (NSW), which are often under-represented in research.
- The perspectives of patients, carers, and clinicians will be captured during data collection.
- Researchers, patient representatives, and health service providers will actively collaborate to ensure research is clinically relevant and meaningful to the community.
- Recruitment will be restricted to patients, carers, and clinicians enrolled in the NSW Patient-Reported Measures (PRMs) Health Outcomes and Patient Experiences (HOPE) programme. We may not capture the experiences of patients, carers, and clinicians participating in other PRMs initiatives.

## Introduction

 Patient-reported outcomes (PROs) are direct reports from patients about their health status, without interpretation by anyone else.^1^ Commonly assessed PROs include symptoms, treatment side effects, functioning and health-related quality of life (HRQL).^2^ PRO measures (PROMs) are questionnaires that quantify PROs with numerical scores^3^ and can be used to improve patient–clinician communication,^4^ monitor responses to treatment^5^, and track symptoms and HRQL over time. PROMs serve to complement biomedical evaluations and provide a holistic view of a patients’ HRQL.

There is growing global enthusiasm for the use of PROMs as healthcare services strive towards a person-centred model of care. This model of care actively engages patients in clinical decision-making^6^ and is an enabler for value-based healthcare- where value is measured by the health outcomes of patients, particularly the quality and safety of their care.^7^ New South Wales (NSW) Health launched the Leading Better Value Care (LBVC) programme in 2017 to build a sustainable health system that prioritises what matters to patients and the community, is personalised, and digitally enabled. The LBVC programme identifies and scales evidence-based initiatives in healthcare delivery and uses rigorous measures and evaluation to show the impact of these initiatives on health outcomes. The aim of the LBVC programme, through the routine collection and use of patient-reported measures (PRMs), is to change the patient–clinician conversation from ‘what’s the matter *with* me?’ to ‘what matters *to* me?’.

A way to shift this patient–clinician conversation is through the collection of PRMs in routine care. NSW Health collects PRMs, including PROMs and patient-reported experience measures (PREMs). As implied by their name, PREMs focus on the patient’s experience with care, complementing the focus of PROMs on patient outcomes. The Agency for Clinical Innovation (ACI), a pillar of NSW Health, facilitates PRMs co-design, implementation, collection, and use *via* a digital platform called Health Outcomes and Patient Experience (HOPE). HOPE enables the collection of PRMs data at the point of care *via* personal computers, tablet devices or smartphones. The data collected are reported in real time to clinicians to support shared decision-making about care and health interventions. In partnership with eHealth NSW, implementation of HOPE commenced in February 2021 and is being implemented by multidisciplinary healthcare teams and across various stages of a patient’s care journey (e.g., when newly diagnosed, when hospitalised with a health event related to a chronic condition, at regular intervals during care) in all local health districts (LHDs) in NSW. LHDs are networks of public hospitals, clinics and community health services funded by the state government.

Recognising the importance of generating new knowledge and optimising the use of PROMs specifically, the University of Sydney and NSW Health developed a research partnership called PROM Presentation, Acceptability, Timing and Health service use in NSW (PROM-PATH). PROM-PATH is a collaboration between researchers, clinical experts and health service managers and is funded by a Partnership grant from the National Health and Medical Research Council (NHMRC).

The NSW PRMs programme was not set up for research but rather to incorporate PRMs into everyday clinical practice by enabling patients and multidisciplinary healthcare teams to capture, review and act on data in a timely and holistic way. Implementation was informed by Accelerating Implementation Methodology^8^ and Behaviour Change Wheel Approach.^9^ There were several planning meetings over the last decade before and during the time HOPE was implemented in a phased approach. ACI has evaluated and revised the system over the years using routine feedback from multidisciplinary healthcare teams, consumers, carers, PRMs experts and internal evaluations.

The PROM-PATH partnership is guided by two key frameworks: the Consolidated Framework for Implementation Research^10^ for health system-level implementation and the normalisation process theory (NPT)^11^ for understanding barriers and facilitators to translation at the individual clinician/patient level.

The life expectancy of Australians is increasing due to advances in healthcare and changes in lifestyles.^12^ However, the HRQL in these extra years of life does not match this successful longevity. The number of Australians with complex chronic conditions continues to grow,^13 14^ which is straining primary care.^15^ In NSW, high-priority chronic conditions in the LBVC programme, that are the focus of this study, include chronic obstructive pulmonary disease (COPD), chronic heart failure (CHF), diabetes, chronic kidney disease, osteoporosis and osteoarthritis. These conditions are also some of the most highly reported in the Australian census.^16^ The collection and use of PROMs is inconsistent in the routine care of Australians with these conditions. Our study will be part of the first state-wide programme of research to rigorously examine the collection of PROMs in HOPE for these chronic conditions. We will recommend improvements to be implemented in the next phase of the NSW PRMs programme.

To enhance our research about patient acceptability, our study will capture the voices and experiences of patients who are Culturally and Linguistically Diverse (CALD), an often-underrepresented population in health research.^17^ CALD refers to people whose first or primary language is not English, or people born overseas in countries that have distinctly different cultures than Australians. A deeper understanding of the CALD experience with using PROMs will be an important contribution to the next phase of the NSW PRMs implementation.

About 29% of Australia’s population are migrants, with over 200 languages spoken at homes,^18^ making us one of the most culturally diverse countries in the world.^19^ CALD communities experience poorer survival outcomes and HRQL.^20^ These outcomes could be due, in part, to the language and cultural barriers CALD patients report when communicating with their healthcare teams,^21 22^ and fear and lack of understanding of a foreign health system.^21^ The NSW PRMs programme aims to be inclusive by offering a translation of PRMs in 10 languages to facilitate PROMs completion in CALD populations.

In research settings, few studies report recruiting CALD populations^23^,^25^ because eligibility criteria often exclude patients with limited English proficiency.^26^ Poor completion rates of PROMs are also reported^27^ potentially due to time and financial barriers to obtain and implement culturally and linguistically appropriate translations. If PROMs are not completed by these populations, patient-reported health outcomes cannot be used to guide shared decision-making with them.

**Figure 1:**
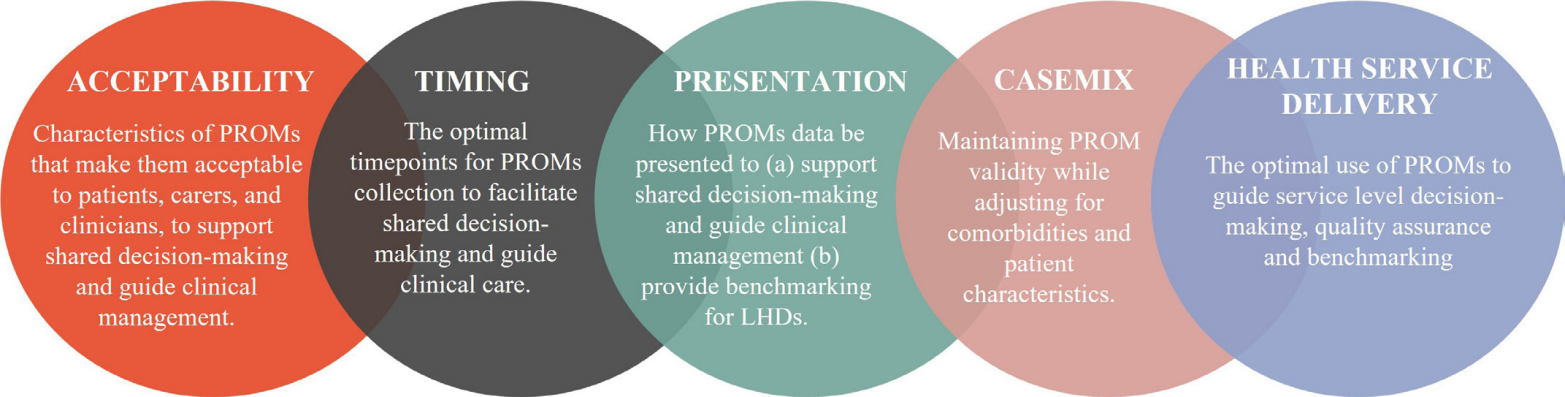
The overarching themes guiding the PROM-PATH research programme, a collaboration between the University of Sydney and New South Wales Health. Abbreviations:local health districts (LHDs); patient-reported outcome measures (PROMs); PROM Presentation, Acceptability, Timing and Health service use in New South Wales (PROM-PATH)

**Figure 2:**
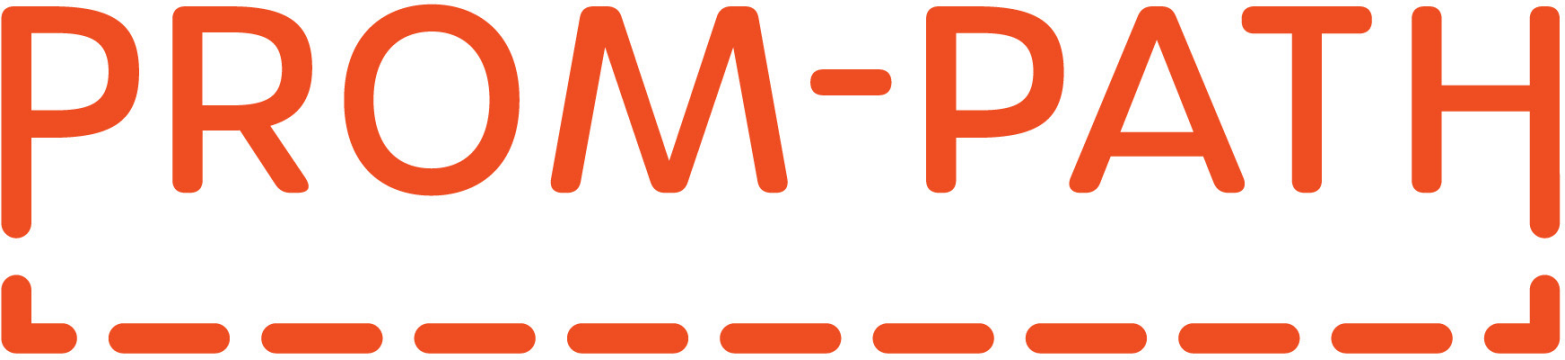
PROM-PATH project logo. Abbreviation: Patient-Reported Outcome Measures Presentation, Acceptability, Timing and Health service use in NSW (PROM-PATH)

This protocol addresses two key research themes in the PROM-PATH programme, developed in collaboration with University of Sydney researchers, the ACI and NSW Ministry of Health (figure 1). Patients, carers and clinicians will be involved to explore (1) the acceptability of PROMs in shared decision-making and (2) appropriate timing of PROMs collection. Our project logo is provided in figure 2.

### Theme 1: Acceptability

The acceptability theme addresses the completion of PROMs by patients, and the use and interpretation of PROM data by clinicians. Acceptability explores whether the content of PROMs is relevant to patient experience and symptoms; the mode of administration is practical; the data are actionable and allocated PROMs are fit-for-purpose for different chronic conditions. If patients find PROMs acceptable, they are more likely to complete PROMs, enabling real-time feedback from patients to inform clinical encounters. In the existing literature, there has been limited focus on understanding whether patients carers, and clinicians believe the content of assigned PROMs accurately captures symptoms, treatment effects and HRQL according to the specific needs and disease trajectories of different chronic conditions. Further, we aim to capture the experience of patients who have been invited to complete PROMs in the HOPE platform but have declined to participate. Herein, these patients will be referred to as PROMs non-completers. These patients can provide valuable insights about how we can better engage and communicate the benefits of PROMs to patients when inviting them to HOPE.

### Theme 2: Timing

The timing theme will examine when to administer PROMs in clinical practice settings and use PROM data for individual patient decision-making. PROMs are ideally completed before appointments or during an episode of care, either in a clinic or at home, so that clinicians can use the data during the encounter. NSW Health engaged clinicians, researchers and experts to determine appropriate standardised schedules for PROMs to be sent to patients via HOPE. Clinicians can override the recommended PROM collection schedule to suit the needs of each patient. It is important to assess if current automated schedules are suitable in everyday care and to understand why clinicians choose to override recommended schedules.

Across the key chronic conditions included in this project, there are variations in symptom and disease trajectories particularly in the experience of COPD and CHF patients. Patients with experience exacerbations in symptoms,^28^ contrasting the steady decline of symptoms for CHF patients. PROMs collected at clinically informative time points enable disease-specific and patient-specific decisions to be made. If reporting of symptoms and HRQL does not occur at clinically meaningful times, patients might not see the value of reporting their PROs.^29^ PROM completion at suboptimal time points may also lead to clinicians making inaccurate conclusions about the benefits and harms of the therapeutic options they provide.

Considering the available evidence and areas that require further investigation, this study asks:

#### Research questions

1. What makes PROMs acceptable to patients with chronic health conditions, including patients from CALD backgrounds?
2. What makes PROMs acceptable to carers who assist patients with chronic health conditions, including patients from CALD backgrounds?
3. What makes PROMs acceptable to clinicians treating patients with chronic health conditions, including patients from CALD backgrounds?
4. Are the current schedules for PROM collection suitable for patients, carers and clinicians?

#### Research aims

1. To understand what makes PROMs acceptable to patients with COPD, CHF, chronic kidney disease, diabetes, osteoporosis and/or osteoarthritis.
2. To understand what makes PROMs acceptable to carers, who are assisting patients with COPD, CHF, chronic kidney disease, diabetes and/or osteoarthritis, to complete PROMs.
3. To determine what makes PROMs acceptable to clinicians treating patients with COPD, CHF, chronic kidney disease, diabetes, osteoporosis and/or osteoarthritis.
4. To determine informative PROM assessment time points for patients with COPD and/or CHF and if they align with current recommended administration time points. These two chronic conditions were chosen due to their distinctly different disease trajectories.

## Methods and analysis

### Study design

This reporting of this study’s results will follow the COnsolidated criteria for REporting Qualitative research checklist (online supplemental file 1).^30^ This study will be qualitative, using semistructured interviews with purposively sampled participants. Semi-structured interviews will allow patients to raise the concerns relevant to them, without constraint by the interviewer. Data collection will take a qualitative descriptive approach based on the philosophy of naturalistic enquiry^31^ to enable the development of themes about the participants’ experience in using PROMs and enhance understanding of their actions and motivations to complete PROMs.

### Setting

This study will leverage existing professional relationships and infrastructure established by NSW Health and the ACI. Since February 2021, HOPE has been used by inpatient and outpatient healthcare centres across rural, regional and metropolitan NSW, collecting information from patients about their health-related experiences and outcomes using a suite of validated generic HRQL (eg, PROMIS-29) and condition-specific (eg, Oxford Knee Score Survey) PROMs.

### Eligibility criteria

The clinical cohorts of interest are:

- Management of Osteoarthritis (OACCP)
- Osteoporotic Re-fracture Prevention (ORP)
- Chronic Heart Failure (CHF)
- Chronic Obstructive Pulmonary Disease (COPD)
- Renal Supportive Care (End Stage Kidney Disease) (RSC)
- Diabetes High Risk Foot Services (HRFS)
- Inpatient Management of Diabetes Mellitus (IDM)

Initial screening will include patients and carers who meet eligibility criteria in the past 6 months. If required, subsequent screenings will be conducted every 8 weeks until recruitment targets have been met.

Patients and carers enrolled in the NSW PRMs programme, who meet the following eligibility criteria, will be invited to this study:

- Understand and speak at least one of the following languages: English, Mandarin, Cantonese, Vietnamese, Arabic, Greek or Spanish *and*
- 18 years of age or over *and*
- have completed or have been assigned, a PROM within the NSW PRMs Programme, enabled by HOPE *or*
- have assisted a patient to complete PROM(s) (e.g., reading questions and responses aloud) within HOPE as a carer/caregiver/family (i.e., not a proxy completion) *and*
- are enrolled in one or more of the clinical cohorts of interest *and*
- able to give their written informed consent to take part.

Clinicians enrolled in the NSW PRMs programme are eligible to participate if they meet the following criteria:

- Understand and speak English *and*
- 18 years of age or over *and*
- have used HOPE to assign or review PROMs *and*
- are a specialist or clinical staff member involved in the care of patients enrolled in one or more of the clinical cohorts of interest *and*
- able to give their written informed consent to take part.

### Sample

We anticipate a patient sample size of ~50-75 (~5–10 per language group, non-specific targets across chronic conditions), ~10–20 carers and a clinician sample size of ~18. We will monitor recruitment closely and will update participating LHDs regarding the clinical cohorts and/or language groups that need a greater number of participants. Recruitment will continue until thematic saturation is achieved, which we define as being able to understand and explain the patient and clinician experience with no new explanations for choosing to complete or not complete PROMs.^32^ Existing guidance suggests that to reach saturation, 15–20 homogeneous interview participants should be interviewed,^33^ and for larger studies such as our patient population, an upper limit of 50 participants is likely sufficient.^34^

### Recruitment and consent

#### Patients and carers

PRM Project Officers at the ACI will screen patients and carers on HOPE against eligibility criteria every 8 weeks until recruitment targets are met. This ensures that patients who are joining HOPE throughout the study period are invited. The ACI-PRM team will email or mail eligible patients (1) a study invitation and attach a participant information sheet that explains the rationale, design and personal implications of the study and (2) link to an electronic consent (eConsent) form via REDCap, the university’s secure way of capturing personal information for studies. Participants can ask the named University of Sydney researcher any questions and complete the consent form if they choose to participate. The researcher will monitor the REDCap database and contact participants to schedule an interview.

The same recruitment and consent process will occur for CALD participants. Healthcare Interpreters who are employed by NSW Health will be used throughout the study to help translate study documents. During interviews, the researcher will conduct the interview in English. The interpreter will be present to interpret responses between researcher and the CALD participant. There will be Mandarin, Cantonese, Vietnamese, Arabic, Greek and Spanish interpreters available.

The ACI will not share any details about the eligible patients who were contacted about the study by University of Sydney researchers. Likewise, the details of patients who consent and participate in the study will not be shared with any the ACI or NSW Health staff. So that clinics are aware of the study, in the event patients ask their healthcare provider, NSW Health clinics will have access to a summary of the study with the researchers contact information on their closed SharePoint site. This recruitment process ensures NSW Health, the ACI, clinics and healthcare teams do not know if a patient has consented and participated in the study, minimising the risk of coercion.

#### Clinicians

PRM Leads, who are responsible for overseeing PRM rollout in their LHD, will distribute the study invitation and participant information sheet to eligible clinicians within their districts. The invitation will include a link to an eConsent form (via REDCap). It is up to the clinician to decide whether to take part in the study and to complete the eConsent form. If clinicians have any questions or concerns, they may directly contact the named study researcher.

#### Scheduling interviews

For patients, carers, and clinicians, University of Sydney researchers will monitor eConsent via the REDCap database and contact participants (by telephone or email) who have consented and provided contact details. Researchers will provide a detailed explanation of the study, answer any questions participants might have, schedule a time for the interview and build initial rapport with participants. Researchers will send a follow-up email including the date, time and location of the interview, and a copy of the PROMIS-29 (for their reference), which is the PROM currently completed by most patients in HOPE.

### Interviews

A one-off interview will be conducted by a researcher over Zoom (which is a University-supported platform), or telephone and may last between 30 and 60 min. The researchers’ will be experienced with qualitative research methods and/or be a consumer representative and/or identify as CALD. Including options for digital methods for interviews will help include patients living in rural and remote areas of NSW, who may not be able to travel to metropolitan Sydney (where researchers are based) due to cost, time and/or ill health. Telephone interviews may also help patients feel more anonymous and, therefore, comfortable sharing their experiences in-depth with the researcher. If feasible, some CALD interviews may be conducted in person.

Study researchers will use an interview guide (online supplemental file 2), guided by Normalisation Process Theory,^11^ and developed and piloted with consumer representatives and the broader project team. At the start of the interview, the PROMIS-29 will be referred to by the interviewer to ascertain if the patient recalls completing this PROM. Participants who are recruited as patients or carers will be asked questions about whether they believe the content of their assigned PROMs is appropriate to their lived experience with their condition; preferred timing for PROM completion; and for patients who identify as CALD, if PROMs are culturally appropriate and if there are any specific barriers or enablers to PROM completion. For PROMs non-completers, we will ask a different set of questions related to why patients and/or carers opted out of PROM completion. Clinicians will be asked to reflect on their use of PROMs during routine care and whether assigned PROMs are appropriate for their patients’ disease trajectory and if the data are actionable in the context of managing chronic conditions.

All interviews will be audio recorded and transcribed using Zoom and/or Microsoft Stream. Study researchers will manually check transcripts alongside audio after the interview. Study researchers will take notes during and after interviews to mark non-verbal cues and thoughts. All transcripts will be deidentified and participants will be assigned a study ID for analysis and a pseudonym for reporting.

### Analysis

One researcher will analyse interviews, and a subset of data will be analysed by a second study researcher with expertise in qualitative research methods. These researchers will consult the wider study team, comprising a range of clinical and PROM implementation expertise, to ensure analysis is participative and iterative.

Transcripts will be coded using NVivo V.12. An inductive coding approach will be taken, which is appropriate as 1) we seek to evaluate the existing NSW PRMs roll-out and 2) PROMs use is still in its infancy in some CALD populations. The analysis will be governed by Normalisation Process Theory (NPT), which examines the extent to which interventions (like PROMs) are a routine, or ‘normal’, part of primary care.^35^ NPT bridges the gap between research (e.g., PROMs as endpoints in clinical trials) and implementation into new contexts (i.e., PROMs in routine care of chronic conditions). An initial coding framework will be developed using NPT (deductive coding), with additional codes generated based on the interview data (inductive coding). Depending on emerging themes in the data, we may include subgroup analyses to compare findings across languages and/or chronic conditions.

Four key components of NPT will be considered in the composition of codes for thematic analysis:^35^

1. Coherence: Do PROMs and their benefits make sense for participants?
2. Cognitive participation: How do participants engage with PROMs and use the HOPE platform?
3. Collective action: What have participants done to enable PROMs to work in routine care?
4. Reflexive monitoring: How do participants judge the benefits of PROMs?

### Patient and public involvement

BR and MF are consumer representatives for the overall research programme—PROM-PATH. They are involved in: all planning stages, developing and approving patient materials for this study, and ensuring study outcomes are relevant and applicable to the broader community. During study design, both representatives advocated for an inclusive study that welcomes people with all lived experiences and helped assess participants’ time commitment. BR and MF will co-develop a strategy to disseminate findings to patients, carers and families. This will include choosing which results to share and the most appropriate formats to convey information clearly. We will report patient and public involvement in this research using the GRIPP2 framework.^36^

### Ethics approval

Ethics approval was obtained by the University of Sydney Human Ethics Committee (Study Protocol #X24-0138). Governance authorisations will be obtained for each LHD who opts in to the study. Opt-in will be obtained via a form completed and returned to University of Sydney researchers.

### Ethics and dissemination

The study will be conducted in accordance with the principles of Good Clinical Practice as detailed by the Australian Health Safety Regulation (2006) and the Australian Code for the Responsible Conduct of Research (2007) by adhering to the study protocol. Data will be securely archived for 5 years, which is recommended in the Management of Data and Information in Research by the NHMRC. Five years post study completion, electronic files will be permanently deleted from the file server. Regular project team meetings will be held to monitor recruitment rates, study timelines, data quality and ensure compliance.

At the conclusion of the study, reports will be submitted to the ethics committee, NSW Health and the NHMRC. If study participants opt to receive study results when they complete the consent form, they will receive results in the form of a plain-language summary. Findings will also be presented in de-identified form at conferences and published in scientific peer-reviewed journals. We will collaborate with our consumer representatives (BR and MF) to design a dissemination strategy for consumers outside the study.

## supplementary material

10.1136/bmjopen-2023-083346 online supplemental file 1

10.1136/bmjopen-2023-083346 online supplemental file 2
