# Supplementary material for: Acceptability and timing considerations when administering patient-reported outcome measures (PROMs) among people with chronic health conditions who are culturally and linguistically diverse (CALD): a qualitative study protocol
Source: BMJ Open. 2024 Sep 12;14(9):e083346. doi: 10.1136/bmjopen-2023-083346 (PMC11404136; doi:10.1136/bmjopen-2023-083346)
Supplement: online supplemental file 2 [file bmjopen-14-9-s002.pdf]

Supplementary File 2: Indicative interview guide for Theme 1 (Acceptability) and Theme 2 (Timing)

1. **Introduction**
2. **Main interview questions:**

Patients

- For patients who identify as culturally and linguistically diverse (CALD): questions will be framed in relation to their cultural context and experiences with navigating English and/or translated PROMs
- Determining if the patient completed PROM before, or if opted-out
    - If completed before:
        - Experience in completing PROM
        - Use of PROM data
        - Perceptions of the optimal time to complete PROMs
    - If opted-out or forgotten completing PROM
        - Probing why (e.g. busy)
        - Information provided about the PROM by healthcare staff
        - What would make it easier to complete PROMs in future
        - Perceived challenges of completing PROMs
        - Perceived benefits of completing PROMs
- Demographic questions: Gender, age, highest level of education, occupation, employment status (e.g. full, time, part-time), living situation, the main language spoken at home, ethnicity (self-described), generation living in Australia, country of birth

Carers

- Experience in helping a patient to complete PROM(s)
- First impressions of the content of PROMs (e.g. cultural suitability)
- Ways PROMs data used
- Perceptions around the timing of PROM completion
- Demographic questions: Gender, age, highest level of education, occupation, employment status (e.g. full, time, part-time), living situation, years of supporting a person with a chronic condition, relationship to the person with a chronic condition, any chronic conditions, the main language spoken at home, ethnicity (self-described), country of birth, postcode.

Clinicians

- Professional background
- Experience in working with patients from an identified clinical cohort
- PROMs used, rationale for use, and experience in using PROMs
    - Probes: Perceived benefits and challenges of using PROMs
- Understanding disease trajectories and how administration of PROMs can be linked to clinical events
- Experience using PROMs in culturally and linguistically diverse populations
- Demographic questions [time permitting]: ethnicity (self-described), country of birth, age

3. **Open-ended concluding questions**
4. **Conclusion of interview**
