# Supplementary material for: Acceptability and timing considerations when administering patient-reported outcome measures (PROMs) among people with chronic health conditions who are culturally and linguistically diverse (CALD): a qualitative study protocol
Source: BMJ Open. 2024 Sep 12;14(9):e083346. doi: 10.1136/bmjopen-2023-083346 (PMC11404136; doi:10.1136/bmjopen-2023-083346)
Supplement: Supplementary file 3 [file bmjopen-2023-083346.reviewer_comments.pdf]

PEER REVIEW HISTORY

BMJ Open publishes all reviews undertaken for accepted manuscripts. Reviewers are asked to complete a checklist review form and are provided with free text boxes to elaborate on their assessment. These free text comments are reproduced below.

ARTICLE DETAILS

**TITLE (PROVISIONAL)**	Acceptability and timing considerations when administering patient-reported outcome measures (PROMs) among people with chronic health conditions who are culturally and linguistically diverse (CALD): a qualitative study protocol.
**AUTHORS**	Nikolovski, Jessica; Morton, Rachael; Mercieca-Bebber, Rebecca; Armstrong, Matilda; Hartas, Gill; Rossiter, Brad; Fagan, Margaret; Tinsley, Melissa; Snyder, CF; Aiyegbusi, Olalekan Lee; Amin-Korim, Rubina; Sutherland, Kim; Rutherford, Claudia

VERSION 1 - REVIEW

**REVIEWER NAME**	***Kamran, Rakhshan***
**REVIEWER AFFILIATION**	University of Oxford Nuffield Department of Orthopaedics Rheumatology and Musculoskeletal Sciences
**REVIEWER CONFLICT OF INTEREST**	Na
**DATE REVIEW RETURNED**	02-Feb-2024

**GENERAL COMMENTS**	This article presents the protocol for a qualitative study aiming to investigate acceptability and timing of PROMs in NSW Health collected and used at point of care via the HOPE platform. The protocol outlines that semi-structured interviews will be conducted with patients and clinicians virtually, via telephone, or in person. As this study is collecting perspectives from CALD communities and those who may be under-represented in research, this is an important study, with results which can aid other researchers to potentially understand how to better incorporate diverse participants in their projects. This study is also looking at practical components of PROM implementation, specifically, acceptability and timing - which are salient and relevant factors to investigate. This is an interesting protocol and I recommend publication with the following minor revisions:<br><br>1. Introduction, page 7, line 10: It is reported that HOPE is being implemented across all LHDs in NSW. It is later reported that PROMs will be routinely collected and used in HOPE (line 45). I am wondering if any of your prior implementation work was guided with a theory/model/framework-informed approach, using guidance from implementation science? Please briefly explain and provide an overview of how implementation of HOPE was operationalised. This could be useful for the readership interested in undertaking their own implementation work involving digital platforms.<br><br>2. Introduction, page 8: Please explain how you define acceptability, and which components of acceptability you are particularly interested in.<br><br>3. Methods, page 12, line 50: How was this sample size estimate

	derived?<br><br>4. Methods, page 15: It is mentioned that inductive coding will be undertaken, governed by NPT. I agree that NPT is particularly relevant for PROM implementation as it aims to understand how an innovation becomes routinised. However, please could you explain in a bit more detail how you will balance an inductive coding approach with the "fixed" key components of NPT?<br><br>5. Patient and public involvement: Please explain that you will follow GRIPP2 reporting guidelines to report PPI.

**REVIEWER NAME**	***Stern, Brocha***
**REVIEWER AFFILIATION**	Icahn School of Medicine at Mount Sinai
**REVIEWER CONFLICT OF INTEREST**	Na
**DATE REVIEW RETURNED**	16-Feb-2024

**GENERAL COMMENTS**	I appreciate the opportunity to review this interesting paper on a very timely and important topic. This is a protocol for a qualitative study of clinician and patient perspectives of acceptability and timing of patient-reported outcome measures collected in routine care for chronic conditions. I particularly value the inclusion of linguistically and culturally diverse patients. I have a few comments that may help with clarity of reporting. [Page numbers refer to the authors' page numbers.]<br><br>• Abstract: P. 4, lines 20-201: Is this about determining the existing "timing" or preferences for/acceptability of timing?<br>• Abstract: P. 4, lines 30-31: Here, the sampling plan appears to be language x condition, which would mean many more participants than 75 if there are even just 5 per cell since there are 6 languages and 7 conditions. In contrast, p. 12, lines 50-54 just indicates purposeful sampling across the 6 languages.<br>• Abstract: P. 4, lines 35-41: Will patients be directly asked about cultural barriers? Or is this something the researchers will infer perhaps from comparing barriers between the English-speaking and CALD participants? More generally, these barriers aren't discussed elsewhere in the paper despite potentially being quite important and a more unique contribution of this study.<br>• General: The authors report that PROMs are collected across the care continuum, but the context could still be more clearly specified. Are these collected by physicians across the continuum? Multidisciplinary clinicians? Is there an anchor that starts off the continuum (e.g., a hospitalization related to one of these conditions)?<br>• P. 8, "Theme 1: Acceptability" – a) line 60: should it also refer to use by patients since the graphic talks about shared decision making? b) Inclusion of the non-completers is very valuable. But reasons for non-completion likely extend far beyond characteristics of the measure, which is the focus of acceptability in the graphic. Will the conversation with these participants focus on measure characteristics?<br>• P. 10, Lines 36-41: Questions about timing will only be asked for participants in these two clinical groups? Also, "most informative" may be challenging to truly ascertain with just qualitative data. This seems to be about preferences and perspectives of optimal timing.<br>• P. 11, lines 42-43: What does it mean to have assisted a patient

	with completion? Is this a caregiver/carer/partner – will they also be eligible for enrollment?<br>• P. 12, lines 42-43: How will the authors know before enrollment that the person can share their experiences in a thoughtful way? And this seems quite subjective.<br>• P. 12, lines 57-59: What does achieving data saturation "for each chronic condition and language" mean? Is this per unique cell? E.g., CHF Vietnamese? Can the authors even guarantee 1 patient per cell? They would need a larger sample for that. Or are the authors saying they will look across each condition regardless of language (e.g., CHF) and then separately look across language regardless of condition (e.g., Vietnamese)? And how are clinicians factored into saturation?<br>• P. 12, line 53: So the authors will purposely enroll 1 clinician per LHD? Will this be enough to ensure different professional/specialty voices?<br>• Role of the PROMIS-29 (p. 14, lines 37-40: Will this be sent to all patients even those who are not being assigned this measure? How will it be used during the interview?<br>• P. 15, lines 10-22: These topics/questions don't directly align with previous topics mentioned, such as timing, etc. It would be helpful to the reader if the author more directly aligned the different sections, from the research questions, interview topics, data analysis, etc.<br>• P. 15, Data analysis: Will there be any subgroup analyses? E.g., Comparisons between different groups of participants to look at differences by conditions, languages, etc.?<br>• General: Given the authors' interest in culturally and linguistically diverse individuals, what is missing from this paper is a deeper engagement with the literature regarding cultural/ethnic/linguistic considerations for PROM selection, collection, and use. I would encourage the authors to look at some of the literature that talks about topics like equity, equality, disparities, literacy, culture, etc. For example, doi: 10.1007/s11136-021-02999-8 | doi: 10.3899/jrheum.141168 – I am not necessarily suggesting these specific references for this protocol, but there has been a growing amount of literature on this topic over the past few years that should be considered.

VERSION 1 – AUTHOR RESPONSE

REVIEWER 1 FEEDBACK	RESPONSE
**This article presents the protocol for a qualitative study aiming to investigate acceptability and timing of PROMs in NSW Health collected and used at point of care via the HOPE platform. The protocol outlines that semi-structured interviews will be conducted with patients and clinicians virtually, via telephone, or in person. As this study is collecting perspectives from CALD**	**Thank you for reviewing and providing feedback on our protocol manuscript. We appreciate your interest in our research, and your recommendations have helped strengthen this manuscript. We have addressed each of your points in this letter and tracked changes in the manuscript.**

**communities and those who may be under-represented in research, this is an important study, with results which can aid other researchers to potentially understand how to better incorporate diverse participants in their projects. This study is also looking at practical components of PROM implementation, specifically, acceptability and timing – which are salient and relevant factors to investigate. This is an interesting protocol and I recommend publication with the following minor revisions:**	
**1. Introduction, page 7, line 10: It is reported that HOPE is being implemented across all LHDs in NSW. It is later reported that PROMs will be routinely collected and used in HOPE (line 45). I am wondering if any of your prior implementation work was guided with a theory/model/framework-informed approach, using guidance from implementation science? Please briefly explain and provide an overview of how implementation of HOPE was operationalised. This could be useful for the readership interested in undertaking their own implementation work involving digital platforms.**	**Thank you for your interest in HOPE, and we agree that it is a fantastic example of implementing digitally enabled patient-reported outcome measure (PROM) completion. On page 6, paragraph 2, we added the following:**<br><br>**"The Agency for Clinical Innovation (ACI) Patient Reported Measures (PRM) program was not set up for research but rather to incorporate PRMs into everyday clinical practice by enabling patients and multidisciplinary care teams (MDTs) to capture, review and act on data in a timely and holistic way. Implementation was informed by Accelerating Implementation Methodology (AIM)[1] and Behaviour Change Wheel Approach[2]. There were also several planning meetings over the last decade before and during the time HOPE was implemented in a phased approach. ACI have evaluated and revised the system over the years using routine feedback from MDTs, consumers, carers, PRM experts and internal evaluations.**<br><br>**The PROM-PATH partnership is a body of research that aims to strengthen health systems through use of patient-reported outcome measures (PROMs) in New South Wales (NSW) and is guided by two key frameworks: the Consolidated Framework for Implementation Research (CFIR)[3] for health system level implementation; and the Normalisation Process Theory (NPT) [4] for understanding barriers and facilitators to translation, at the individual clinician/patient level. These two frameworks are well suited to our project aims because they facilitate understanding at the individual, service**

	**and system levels and have been used in our previous research."**
**2. Introduction, page 8: Please explain how you define acceptability, and which components of acceptability you are particularly interested in.**	**We have now clarified which components of acceptability (actionability, content, fit-for-purpose) we are interested in on page 8, paragraph 1, under 'Theme 1: Acceptability': "Acceptability explores whether the content of PROMs is relevant to patient experience and symptoms; the mode of administration is practical; the data is actionable and allocated PROMs are fit-for-purpose for different chronic conditions".**
**3. Methods, page 12, line 50: How was this sample size estimate derived?**	**Our sample size estimate is clarified under 'Sample' on page 13, paragraph 1:<br>"We anticipate a patient sample size of ~50 across languages and chronic conditions (~5-10 per language group, non-specific targets across chronic conditions), ~10-20 carers, and a minimum clinician sample size of ~18, which will ensure coverage across the 18 LHDs in NSW. These targets are guidelines as recruitment will continue until data saturation is achieved."**
**4. Methods, page 15: It is mentioned that inductive coding will be undertaken, governed by NPT. I agree that NPT is particularly relevant for PROM implementation as it aims to understand how an innovation becomes routinised. However, please could you explain in a bit more detail how you will balance an inductive coding approach with the "fixed" key components of NPT?**	**Balancing inductive and deductive coding approaches is an important consideration in qualitative research. We have clarified our approach on page 16, paragraph 3: "An initial coding framework will be developed using NPT (deductive coding), with additional codes generated based on the interview data (inductive coding). Depending on emerging themes in the data, we may include sub-group analyses to compare findings across languages and/or chronic conditions."<br><br>Please note the topic guide questions in the qualitative in-depth semi-structured interviews are informed by NPT; for example, questions about implementation processes. The data from these questions and conclusions about the barriers and facilitators can be reported back using NPT constructs as relevant such as Coherence, Cognitive participation, Collective action, Integration and Reflexive monitoring [4], highlighted on page 16-17:**

	1. **"Coherence: Do PROMs and their benefits make sense for patients and clinicians?**<br>2. **Cognitive participation: How do patients and clinicians engage with PROMs and use the HOPE platform?**<br>3. **Collective action: Have patients and clinicians done anything to enable PROMs to work in routine care?**<br>4. **Reflexive monitoring: How do patients and clinicians judge the benefits of PROMs?"**
**5. Patient and public involvement: Please explain that you will follow GRIPP2 reporting guidelines to report PPI.**	**Using GRIPP2 is an excellent recommendation to document patient and public involvement systematically. We have included this framework under 'Patient and Public involvement statement' on page 16, paragraph 1: "We will report patient and public involvement in this research using the GRIPP2 framework[5]".**

**REVIEWER 2 FEEDBACK**	**RESPONSE**
**I appreciate the opportunity to review this interesting paper on a very timely and important topic. This is a protocol for a qualitative study of clinician and patient perspectives of acceptability and timing of patient-reported outcome measures collected in routine care for chronic conditions. I particularly value the inclusion of linguistically and culturally diverse patients. I have a few comments that may help with clarity of reporting. [Page numbers refer to the authors' page numbers.]**	**We thank you for your detailed review of our manuscript. Your thorough feedback has been incorporated into our manuscript and we believe it has further strengthened the rigor of our protocol. We have addressed each of your points in this letter, as well as in tracked changes in the manuscript.**
**Abstract: P. 4, lines 20-201: Is this about determining the existing "timing" or preferences for/acceptability of timing?**	**We have clarified that 'timing' refers to preferences for PROM completion and use as well as the acceptability of the current PROM completion time-points.**<br><br>**Please see**<br>1. **Abstract, page 3, "preferred timing of PROM completion and use"**

	2. Introduction page 9, paragraph 1 "The timing theme will examine when to administer PROMs in clinical practice settings and use PROM data for individual patient decision making"<br><br>3. Methods page 15, paragraph 3: "preferred timing of PROM completion".
Abstract: P. 4, lines 30-31: Here, the sampling plan appears to be language x condition, which would mean many more participants than 75 if there are even just 5 per cell since there are 6 languages and 7 conditions. In contrast, p. 12, lines 50-54 just indicates purposeful sampling across the 6 languages.	Thank you for picking this up. We have now clarified recruitment targets on page 3 (Abstract) and 13, paragraph 1 (Methods): "We anticipate a patient participant sample size of ~50 -75 across languages and chronic conditions (~5-10 per language group, non-specific targets across chronic conditions), ~10-20 carers, and a minimum clinician participant sample size of ~18, which will ensure coverage across the 18 LHDs in NSW. We will monitor recruitment closely and will update districts about the clinical areas that need a greater number of participants. Recruitment will continue until data thematic saturation is achieved for each chronic condition and language, which we define as being able to understand and explain the patient and clinician experience with no new explanations for choosing to complete or not complete PROMs[32]."
Abstract: P. 4, lines 35-41: Will patients be directly asked about cultural barriers? Or is this something the researchers will infer perhaps from comparing barriers between the English-speaking and CALD participants? More generally, these barriers aren't discussed elsewhere in the paper despite potentially being quite important and a more unique contribution of this study.	We agree this is a unique and important contribution. Participants who identify as CALD will be asked specific questions related to cultural acceptability of PROMs and barriers to completion and use of PROMs. These differences may arise when comparing barriers between English-speaking and CALD participants.<br><br>We have included more detail on page 16, paragraph 1: "for patients who identify as CALD, if PROMs are culturally appropriate and suitable and if there are any specific barriers or enablers to PROM completion. For PROM non-completers, we will ask a different set of questions related to why patients and/or carers opted-out of PROM completion".
General: The authors report that PROMs are collected across the care continuum, but the context could still be more clearly specified. Are these collected by physicians across the continuum? Multidisciplinary clinicians? Is there an anchor that starts off the continuum (e.g., a	This is an important distinction you have raised. We have now clarified 'care continuum' as multidisciplinary healthcare teams, as well as when during a patient's care PROMs are collected (e.g., newly diagnosed with a chronic condition, at regular intervals during care, on deterioration of health status) on page 6, paragraph 1:

**hospitalization related to one of these conditions)?**	**"HOPE is being implemented by multidisciplinary healthcare teams and across various stages of a patient's care journey (e.g., when newly diagnosed, when hospitalised with a health event related to a chronic condition, at regular intervals during care) in all local health districts (LHDs) in NSW."**
**P. 8, "Theme 1: Acceptability" – a) line 60: should it also refer to use by patients since the graphic talks about shared decision making? b) Inclusion of the non-completers is very valuable. But reasons for non-completion likely extend far beyond characteristics of the measure, which is the focus of acceptability in the graphic. Will the conversation with these participants focus on measure characteristics?**	**Thank you for raising this point. We agree that the characteristics of the PROM may be a contributing factor for many (e.g. available languages, nature of questions, length, reading level); however, other concerns may include but are not limited to how much time the patient has available to complete the questionnaire, or how concerned they are about data security.**<br><br>**We have added on page 11, paragraph 1: "semi-structured interviews will allow patients to raise the concerns relevant to them, without constraint by the interviewer".**
**P. 10, Lines 36-41: Questions about timing will only be asked for participants in these two clinical groups? Also, "most informative" may be challenging to truly ascertain with just qualitative data. This seems to be about preferences and perspectives of optimal timing.**	**Please note only participants recruited from COPD and/or heart failure clinical cohorts will be asked questions about timing of PROMs (as per our study aims). This is because of their distinct disease trajectories.**<br><br>**We agree that 'most informative' is challenging to address using qualitative research alone. However qualitative methods will facilitate a deeper understanding about the context of PROMs from an individual's perspective in the management of their chronic condition. We have amended to "informative" on page 10, aim 4.**
**P. 11, lines 42-43: What does it mean to have assisted a patient with completion? Is this a caregiver/carer/partner – will they also be eligible for enrollment?**	**We have clarified what assisted completion means on page 12: "have assisted a patient to complete PROM(s) (e.g., read questions and responses aloud) within HOPE as a carer/caregiver/family (i.e., not proxy completion)".**<br><br>**As caregiver/carer/partners are also eligible for recruitment to this study, we have added this into the eligibility criteria on:**<br><br>• **Page 11, paragraph 3:"Patients and carers enrolled in the NSW Health PRMs program,**

	who meet the following eligibility criteria, will be invited to this study” and<br>• Specific research questions and aims on page 9 and 10: “What makes PROMs acceptable to carers who assist patients with chronic health conditions, including patients from CALD backgrounds?” and “To understand what makes PROMs acceptable to carers who are assisting patients with COPD, chronic heart failure, chronic kidney disease, diabetes and/or osteoarthritis to complete PROMs?”
P. 12, lines 42-43: How will the authors know before enrollment that the person can share their experiences in a thoughtful way? And this seems quite subjective.	We agree that this is subjective and hard to measure. We have excluded this criterion from initial screening on page 12 as we cannot identify this feature within our proposed recruitment method of screening the HOPE database.
P. 12, lines 57-59: What does achieving data saturation “for each chronic condition and language” mean? Is this per unique cell? E.g., CHF Vietnamese? Can the authors even guarantee 1 patient per cell? They would need a larger sample for that. Or are the authors saying they will look across each condition regardless of language (e.g., CHF) and then separately look across language regardless of condition (e.g., Vietnamese)? And how are clinicians factored into saturation?	Thank you for picking this up. We have now clarified recruitment targets on page 3 (Abstract) and 13, paragraph 1 (Methods): “We anticipate a patient participant sample size of ~50 -75 across languages and chronic conditions (~5-10 per language group, non-specific targets across chronic conditions), ~10-20 carers, and a minimum clinician participant sample size of ~18, which will ensure coverage across the 18 LHDs in NSW. We will monitor recruitment closely and will update districts about the clinical areas that need a greater number of participants. Recruitment will continue until data thematic saturation is achieved for each chronic condition and language, which we define as being able to understand and explain the patient and clinician experience with no new explanations for choosing to complete or not complete PROMs[32].”
P. 12, line 53: So the authors will purposely enroll 1 clinician per LHD? Will this be enough to ensure different professional/specialty voices?	We aim to have a minimum of 1 clinician per LHD to represent each jurisdiction. However, depending on interest and availability, we may recruit more. We have added more detail on page 13, paragraph 1: “We will monitor recruitment closely and will update districts with regards to the clinical areas that need a greater number of participants.”
Role of the PROMIS-29 (p. 14, lines 37-40: Will this be sent to all patients even those who are not being assigned this measure? How will it be used during the interview?	All patients, even if they have not completed PROMIS-29 (whether not assigned or opted-out by choice), will be provided with a copy of this PROM in their study information pack. This will be referred to at the start of the interview. If patients do not recall completing PROMIS-29, specific interview questions have been developed. We have clarified

	**this further in the manuscript on page 15, paragraph 3: "At the start of the interview, the PROMIS-29 will be referred to by the interviewer to ascertain if the patient recalls completing this PROM. If the patient confirms they have not completed or opted out of completing this PROM, specific interview questions have been developed to understand why this is".**
**P. 15, lines 10-22: These topics/questions don't directly align with previous topics mentioned, such as timing, etc. It would be helpful to the reader if the author more directly aligned the different sections, from the research questions, interview topics, data analysis, etc.**	**On page 15, paragraph 3, we have now clarified interview topics to better align with our research questions and aims:**<br><br>**"Participants who are recruited as patients or carers will be asked questions about: whether they believe the content of their assigned PROMs are appropriate to their lived experience with their condition; preferred timing for PROM completion; and for patients who identify as CALD, if PROMs are culturally appropriate and suitable, and if there are any specific barriers or enablers to PROM completion. For PROM non-completers, we will ask a different set of questions related to why patients and/or carers opted-out of PROM completion. Clinician participants will be asked to reflect on their use of PROMs during routine care and whether assigned PROMs are appropriate for their patients' disease trajectory and if the data is actionable in the context of chronic condition management and treatment."**<br><br>**Please note the research themes of the PROM-PATH project (e.g. Acceptability, Timing) are distinct from the interview topics. We have provided a Supplementary file with the interview guide topics.**
**P. 15, Data analysis: Will there be any subgroup analyses? E.g., Comparisons between different groups of participants to look at differences by conditions, languages, etc.?**	**We have considered different options for sub-group analyses, but in qualitative research, generalisability is often not a goal and is rarely feasible. As interviews commence, we will monitor emerging data and determine if and which sub-groups can be analysed (e.g. contrasting between chronic disease areas). We have added a statement to make this clear on page 16, paragraph 4:" Depending on emerging themes in the data, we may include sub-group analyses to compare findings across languages and/or chronic conditions."**
**Given the authors' interest in culturally and linguistically diverse individuals, what is missing from this paper is a deeper engagement with**	**Thank you for the links to helpful articles. On page 7, paragraph 2 in the Introduction of the manuscript, we have drawn from the literature regarding cultural/ethnic/and linguistic considerations for**

**the literature regarding cultural/ethnic/linguistic considerations for PROM selection, collection, and use. I would encourage the authors to look at some of the literature that talks about topics like equity, equality, disparities, literacy, culture, etc. For example, doi: 10.1007/s11136-021-02999-8 | doi: 10.3899/jrheum.141168 – I am not necessarily suggesting these specific references for this protocol, but there has been a growing amount of literature on this topic over the past few years that should be considered.**	**PROM selection, collection and use: "In research settings, few studies report recruiting CALD populations[6-8] as eligibility criteria often exclude patients with limited English proficiency [9]. Poor completion rates of PROMs are also reported [10], potentially due to time and financial barriers to obtain and implement culturally and linguistically validated PROM translations. If PROMs are not completed by these populations, patient-reported health outcomes cannot be used to guide shared decision-making with them".**

We have made some additional minor changes to improve the clarify of the text:

- **Page 3 'Ethics and dissemination': updated ethics approval to "Sydney Local Health District Human Research Ethics Committee (Study Protocol #2023/697X24-0138)."**
- **Page 12 eligibility criteria addition: "within the NSW PRMs Program, enabled by HOPE"**
- **Page 13 eligibility criteria: "use the HOPE platform to assign or review PROMs" instead of "have administer a PROM using the HOPE platform"**
- **Page 14, paragraph 1: "Mandarin, Cantonese" instead of "Chinese"**
- **Page 17, under 'Ethics and dissemination': "Ethics approval for this study has been obtained from Sydney Local Health District Human Research Ethics Committee (Protocol X24-0138). Governance authorisations will be obtained for each LHD who has opted-in to the study."**

Some additional supplementary file and figures have been added:

- **Supplementary File 2: Interview Topic Guide Outline**
- **Figure 2: PROM-PATH Project Logo**

Additional references provided in this response:

1. McHale, J., T.A. Chick, and E. Miluk, *Implementation Guidance for the Accelerated Improvement Method (AIM)*. 2010, CMU/SEI-2010-SR-032, Software Engineering Institute, Carnegie Mellon University.

2. Michie, S., M.M. Van Stralen, and R. West, *The behaviour change wheel: a new method for characterising and designing behaviour change interventions.* Implementation science, 2011. 6: p. 1-12.

3. Damschroder, L.J., et al., *Fostering implementation of health services research findings into practice: a consolidated framework for advancing implementation science.* Implementation science, 2009. 4: p. 1-15.

4. Nilsen, P. and S.A. Birken, *Handbook on implementation science*. 2020: Edward Elgar Publishing.

5. Staniszewska, S., et al., *GRIPP2 reporting checklists: tools to improve reporting of patient and public involvement in research.* Research Involvement and Engagement, 2017. 3(1): p. 13.

VERSION 2 – REVIEW

**REVIEWER NAME**	***Kamran, Rakhshan***
**REVIEWER AFFILIATION**	University of Oxford Nuffield Department of Orthopaedics Rheumatology and Musculoskeletal Sciences
**REVIEWER CONFLICT OF INTEREST**	Na
**DATE REVIEW RETURNED**	25-Jul-2024

**GENERAL COMMENTS**	This article presents a protocol for a qualitative study to assess acceptability of PROMs across 7 languages and chronic conditions in NSW. This is an interesting paper, and I recommend publication upon addressing a few minor comments.<br><br>Abstract<br>1. Clear abstract - however, if there is space, please add in a few words about the qualitative approach/analysis you plan to undertake<br><br>Strengths and Limitations Section<br>1. Please expand on why the limitation is a limitation (e.g., please expand and explain that this may mean that not all patient voices will be captured to add more depth to this bullet point)<br><br>Introduction<br>1. Please expand a bit with a description on what the LBVC is and what it involves (e.g., are there educational components, is it mainly promotional material?)<br><br>Methods<br>1. Please explain information on reflexivity/positionality of the interviewer.

**REVIEWER NAME**	***Stern, Brocha***
**REVIEWER AFFILIATION**	Icahn School of Medicine at Mount Sinai
**REVIEWER CONFLICT OF INTEREST**	Na
**DATE REVIEW RETURNED**	03-Aug-2024

GENERAL COMMENTS	Thank you to the authors for their thoughtful changes, which have clarified and strengthened the paper. I look forward to seeing the results of this important work.

VERSION 2 – AUTHOR RESPONSE

REVIEWER 1 FEEDBACK	RESPONSE
This article presents a protocol for a qualitative study to assess acceptability of PROMs across 7 languages and chronic conditions in NSW. This is an interesting paper, and I recommend publication upon addressing a few minor comments.	We are grateful for your second review of our manuscript. Your feedback is insightful and has strengthened of manuscript.
Abstract<br>1. Clear abstract - however, if there is space, please add in a few words about the qualitative approach/analysis you plan to undertake	On page 3, lines 16-18, we have added "Interviews will be analysed using a reflexive thematic approach, guided by Normalisation Process Theory."
Strengths and Limitations Section<br>1. Please expand on why the limitation is a limitation (e.g., please expand and explain that this may mean that not all patient voices will be captured to add more depth to this bullet point)	On page 4, lines 14-16, we have expanded this bullet point to include "thus we may not capture the experiences of patients and clinicians participating in other PRM initiatives."
Introduction<br>1. Please expand a bit with a description on what the LBVC is and what it involves (e.g., are there educational components, is it mainly promotional material?)	In addition to the description of LBVC on page 5, we have expanded with the following lines on page 5, lines 15-17 "LBVC identifies and scales evidence-based initiatives in care delivery and uses rigorous measures and evaluation to show the impact of these initiatives on outcomes."
Methods<br>1. Please explain information on reflexivity/positionality of the interviewer.	On page 14, lines 16-18, we have included information about the interviewers' reflexivity/positionality "The researchers' positionality includes experience with qualitative research methods and/or experience as a consumer representative and/or identify as CALD."
REVIEW 2 FEEDBACK	RESPONSE

**Thank you to the authors for their thoughtful changes, which have clarified and strengthened the paper. I look forward to seeing the results of this important work.**	**Thank you for your review and enthusiasm for our proposed study. We appreciate your time and expertise during the revision of our manuscript.**
